# Environmental and host-associated determinants of microbiome variation in the brown dog tick *Rhipicephalus linnaei* in Thailand

**DOI:** 10.1186/s13071-026-07656-y

**Published:** 2026-09-10

**Authors:** Artharee Rungrojn, Kittipong Chaisiri, Janjira Thaipadungpanit, Elizabeth M. Batty, Piyanan Taweethavonsawat, Onrampa Thempachana, Wandee Kongkaew, Serge Morand, Stuart D. Blacksell

**Affiliations:** 1 Mahidol Oxford Tropical Medicine Research Unit, Faculty of Tropical Medicine, Mahidol University, 420/6 Rajvithi Road, Bangkok, 10400 Thailand; 2 Faculty of Tropical Medicine, Mahidol University, 420/6 Rajvithi Road, Bangkok, 10400 Thailand; 3 Parasitology Unit, Department of Veterinary Pathology, Faculty of Veterinary Science, Chulalongkorn University, Bangkok, Thailand; 4 Biomarkers in Animals Parasitology Research Unit, Faculty of Veterinary Science, Chulalongkorn University, Bangkok, 10330 Thailand; 5 Veterinary Research and Development Center (Upper Southern Region), Thungsong, 80110 Nakhon Si Thammarat Thailand; 6 Centre National de La Recherche Scientifique, France–Kasetsart University and Mahidol University, Bangkok, Thailand; 7 Centre for Tropical Medicine and Global Health, Nuffield Department of Clinical Medicine, University of Oxford, Oxford, UK

**Keywords:** *Rhipicephalus linnaei*, Tick, Microbiome, 16S rRNA, Oxford Nanopore sequencing, Thailand

## Abstract

**Background:**

The brown dog tick, *Rhipicephalus linnaei*, is an important vector of veterinary and zoonotic pathogens worldwide. The composition of the tick-associated microbiome may influence tick physiology, adaptation, and vector competence; however, factors shaping the microbiome of *R. linnaei* in Southeast Asia remain poorly understood.

**Methods:**

A total of 131 *R. linnaei* ticks were collected from dogs across five provinces in Thailand, representing different geographical and ecological settings. Full-length bacterial 16S rRNA sequencing was performed using Oxford Nanopore Technology to characterise bacterial community composition. Microbiome diversity and structure were evaluated by geographical region, sampling area (urban versus rural), blood-feeding status, tick sex, and developmental stage using alpha diversity metrics, Bray–Curtis dissimilarities, principal coordinates analysis, PERMANOVA, and ANCOM-BC2 differential abundance analysis.

**Results:**

The microbiome of *R. linnaei* was dominated by *Candidatus* Coxiella mudrowiae, which was detected in all ticks. Significant differences in bacterial community composition were observed between geographical regions, sampling areas, and blood-feeding status. Urban ticks showed lower bacterial diversity than rural ticks, and engorged ticks showed altered microbial diversity compared with feeding ticks. Sex- and developmental stage-associated differences were less pronounced. Differential abundance analysis identified several bacterial taxa associated with ecological and host-related factors. Putative tick-associated bacterial taxa, including *Anaplasma* and *Ehrlichia* species, were identified at low frequency.

**Conclusions:**

This study demonstrates that geography, habitat type, and blood-feeding status are associated with variation in the microbiome of *R. linnaei* in Thailand. The findings provide new insights into the ecological factors shaping tick-associated bacterial communities in Southeast Asia and highlight the utility of full-length 16S rRNA Nanopore sequencing for vector microbiome investigations.

**Graphical abstract:**

**Figure:**
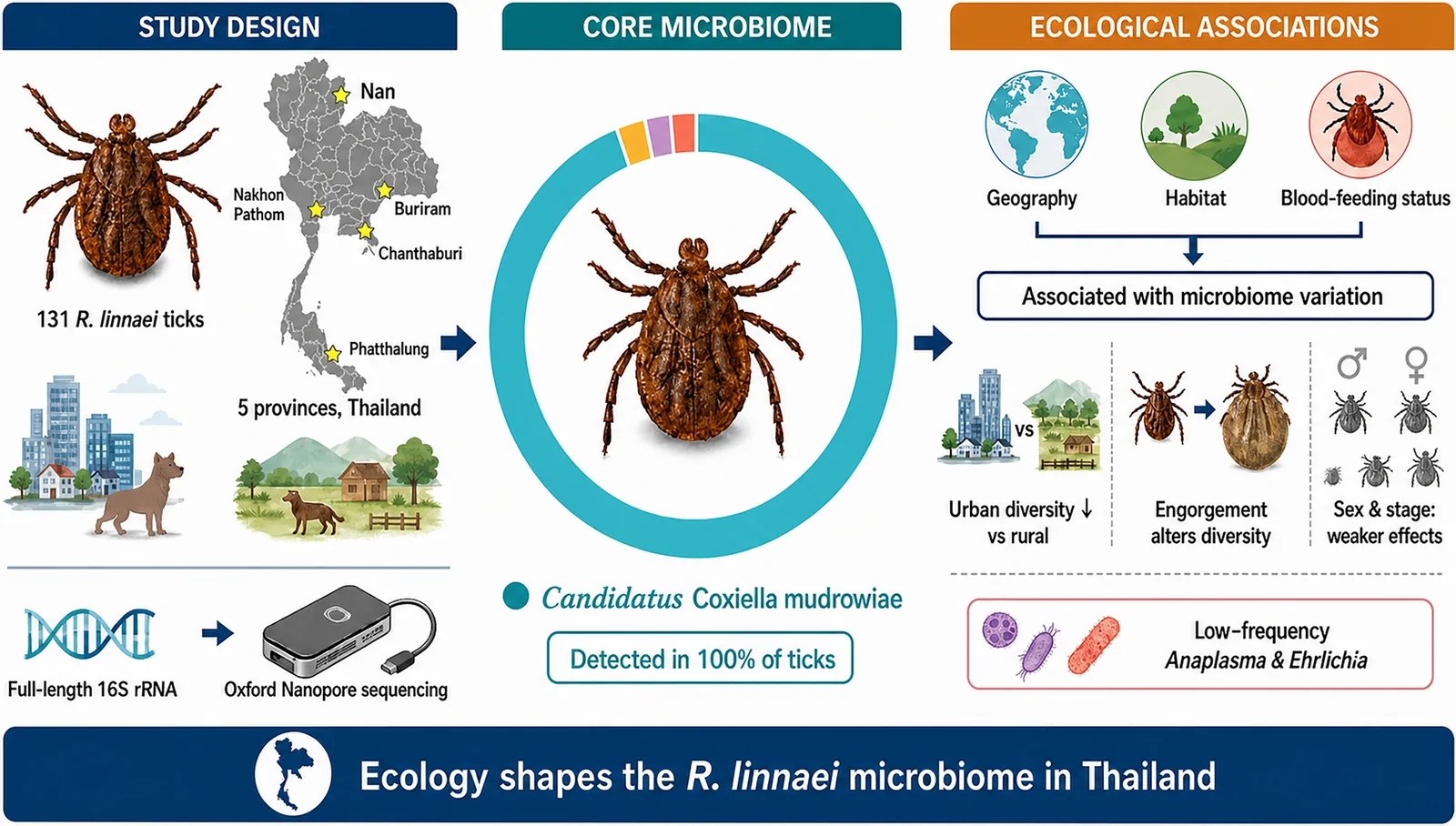

## Background

Ticks are among the most important arthropod vectors of pathogens affecting both human and animal health worldwide, transmitting a wide range of bacterial, viral, and protozoal agents of veterinary and zoonotic importance [1, 2]. Among these, the brown dog tick, *Rhipicephalus sanguineus* sensu lato (s.l.), is one of the most widely distributed tick species globally and is closely associated with domestic dogs in tropical, subtropical, and temperate regions [3]. *Rhipicephalus linnaei* (Audouin, 1826) is a valid species within the former *Rhipicephalus sanguineus* species complex and is the predominant tropical brown dog tick.

[4]. This tick species complex is recognised as a vector of several clinically important pathogens, including *Ehrlichia canis*, *Rickettsia* spp., *Anaplasma* spp., and *Babesia* spp. [5, 6]. Due to the close relationship between dogs and humans, *R. linnaei* is also increasingly relevant within a One Health framework, particularly in rapidly urbanising settings where human companion animal interactions are frequent [7].

In recent years, increasing attention has focused on the microbial communities associated with ticks. The tick microbiome comprises a complex assemblage of symbiotic, commensal, and potentially pathogenic microorganisms that may influence tick physiology, development, reproduction, environmental adaptation, and vector competence [8–10]. Endosymbiotic bacteria such as *Candidatus* Coxiella species are commonly associated with *Rhipicephalus* ticks and are thought to play important nutritional and biological roles within the tick host [11, 12].

Interactions between resident microbiota and tick-borne pathogens may also influence pathogen acquisition, maintenance, and transmission dynamics [10, 13]. Consequently, characterising tick-associated microbial communities is increasingly recognised as important for improving understanding of vector biology and tick-borne disease ecology.

Several intrinsic and extrinsic factors have been shown to shape the tick microbiome. These include tick species, developmental stage, sex, geographical location, host species, environmental conditions, and blood-feeding status [14–16]. Geographical and ecological variation may influence microbial exposure and acquisition through environmental and host-associated pathways. At the same time, blood feeding can substantially alter microbial diversity by introducing host-derived microorganisms and by inducing physiological changes within the tick gut [17, 18]. Despite increasing global interest in tick microbiome ecology, studies from Southeast Asia remain limited, and the bacterial microbiome of *R. linnaei* in Thailand remains poorly characterised.

Recent advances in long-read sequencing technologies have improved the ability to characterise microbial communities at higher taxonomic resolution. Oxford Nanopore Technology (ONT) full-length 16S rRNA sequencing enables near-complete sequencing of the bacterial 16S rRNA gene. It may provide improved species-level classification compared with short-read sequencing approaches targeting partial 16S regions [19, 20]. This approach is increasingly useful for investigations of arthropod-associated microbiomes and microbial ecology.

The present study aimed to characterise the bacterial microbiome of *Rhipicephalus linnaei* ticks collected from dogs across multiple geographical regions of Thailand using full-length 16S rRNA sequencing on an ONT platform. In addition, we investigated the influence of geographical region, sampling environment, blood-feeding status, developmental stage, and tick sex on bacterial community diversity and composition.

## Methods

### Sample collection

Ticks were randomly collected from dogs using forceps in Nan, Chanthaburi, Phatthalung, Buriram, and Nakhonpathom between 2024 and 2025. Ticks were individually placed into 50-mL Falcon tubes and stored at − 80 °C until processing. Tick specimens were identified morphologically using established taxonomic keys [21].

### Molecular species confirmation

For molecular confirmation of species identification, DNA was extracted from 21 systematically selected tick samples and analysed using DNA barcoding of the mitochondrial Cytochrome c Oxidase subunit I (mtCOI) gene [22]. Phylogenetic trees of subsampled tick specimens collected in this study were constructed by MEGA11[23] under the Maximum Likelihood method and the General Time Reversible model with 1000 bootstrap replications.

### Grouping

To analyse the bacterial microbiome in tick samples, we grouped them by geographical region, sampling area, blood meal status, tick stage, and tick sex (Table 1).

**Table 1:** Sample grouping, alpha, and beta diversity

Grouping	Group name	Samples (*n*)	Samples rarefied (*n*)*	Observed	Chao1	Shannon	Bray–Curtis				
							PEMANOVA			Beta dispersion	
				Median (Q1–Q3)	Median (Q1–Q3)	Median (Q1–Q3)	*P* value	R^2^	*F*	*P* value	*F*
Geographical region	Buriram Chanthaburi Nakhonpathom Nan Phatthalung	30 30 6 35 30	30 30 6 31 30	42 (26.25–63.5) 37.5 (29–62) 24.4 (15–32.5) 38 (20.5–56.5) 28.5 (12.5–47.75)	42 (26.25–63.5) 37.5 (29.13–62.25) 24.5 (15–32.5) 38 (20.5–57.5) 28.5 (12.5–47.75)	0.49 (0.25–1.21) 0.83 (0.49–1.23) 0.12 (0.05–0.16) 1.24 (0.81–1.65) 0.12 (0.05–0.46)	0.001	0.145	5.16	0.001	8.87
Sampling area	Rural Urban	65 66	61 66	38 (23–59) 31.5 (18–51.5)	38 (23–59.5) 31.63 (18–51.5)	1.02 (0.59–1.56) 0.25 (0.06–0.69)	0.001	0.089	12.23	0.001	34.02
Blood meal status	Feeding Engorged	97 34	93 34	44 (28–66) 19.5 (9–30.75)	44 (28–66) 19.5 (9–30.75)	0.69 (0.25–1.28) 0.46 (0.06–1.1)	0.007	0.029	3.66	0.055	3.44
Sex	Male Female	40 74	39 73	56 (34.5–95.5) 29 (15–42)	56 (34.5–95.5) 29 (15–42.33)	1.25 (0.45–1.58) 0.4 (0.09–0.95)	0.140	0.015	3.48	0.258	1.19
Stage	Nymph Adult	25 106	23 104	38 (24.5–53) 34 (18–56)	38 (24.5–54) 34 (18–56)	0.93 (0.4–1.2) 0.51 (0.12–1.26)	0.458	0.007	1.48	0.358	0.75

An asterisk (*) indicates the sample size after rarefaction at 50,000 reads

### DNA isolation and sequencing

Total DNA extraction from ticks was performed using the Zymo Pathogen Magbead Kit (Zymo Research, Cat. No R2145) following the manufacturer’s instructions. PCR amplification of approximately 1500 bp of full-length 16S rRNA was carried out using primers 27F and 1492R. The PCR conditions were as follows: PCR was performed in a 25 μL volume, consisting of 0.6 μM of each 16S rRNA forward and reverse primer, 1X SparQ HiFi PCR Master Mix 250R (Quantabio, Beverly, MA 01915), and 1 μL of DNA template (100 ng/μL). The reaction was pre-denatured at 98 °C for 2 min, followed by 34 cycles of 95 °C for 1 min, 61 °C for 1 min, and 68 °C for 3 min (T100 thermal cycler, Bio-Rad, Hercules, CA, USA). Amplicon concentrations were quantified using a Qubit 4 Fluorometer (Thermo Fisher Scientific, Waltham, MA, USA). Following the manufacturer’s instructions, bacterial 16S rRNA gene sequence libraries were prepared using a 16S Native Barcoding kit 1-96 (Oxford Nanopore Technologies, Oxford, Oxfordshire, UK; SQK-NBD114.96). Barcoded libraries were pooled and loaded onto a MinION R10.4 flow cell on a GridION sequencer (Oxford Nanopore Technologies). Sequencing was performed using MinKNOW software (v25.03.7). Base calling was performed using Dorado (v7.8.3) with the SUP model and a minimum Phred quality score >9. Sequencing was performed for 72 h.

### Data cleaning, processing of metagenomic data, and statistical values and diversity in the *R. linnaei* microbiome

Raw ONT reads containing 16S rRNA primer sequences were selected prior to adapter trimming, followed by adapter-trimming using Porechop (v0.2.4) with default parameters [24]. The reads were subsequently filtered using SeqKit [25] to remove sequences with a Phred quality score <10 and lengths outside the 1300–1700 bp range. Read quality was assessed using NanoStat (v1.4.0) with default settings [26]. Taxonomic assignment was performed using EMU with the default database (v3.4.5) [20].

All ONT-generated sequencing data were analysed in R version 4.5.2 [27], with microbial community data structured and managed using the *phyloseq* package [28]. Alpha diversity was characterised through three complementary metrics: observed species richness, the Chao1 richness estimator, and the Shannon diversity index, collectively capturing both the richness and evenness of microbial communities within samples. Pairwise differences in alpha diversity between groups were assessed using the Wilcoxon signed-rank test, with the Holm–Bonferroni correction applied to control the familywise error rate. Beta diversity was quantified using Bray–Curtis dissimilarity matrices to capture compositional differences among microbial communities, and the results were visualised using principal coordinates analysis (PCoA). The statistical significance of between-group differences in community composition was evaluated using Permutational Multivariate Analysis of Variance (PERMANOVA). Differential abundance analysis was subsequently performed using Analysis of Compositions of Microbiomes with Bias Correction 2 (ANCOM-BC2), which accounts for the compositional nature of microbiome sequencing data and provides robust identification of taxa that are significantly enriched or depleted between groups [29].

### Taxonomic assignment limitations

Species-level assignments generated from full-length 16S rRNA sequencing were interpreted cautiously for closely related bacterial taxa, particularly within the genera *Anaplasma* and *Ehrlichia*, where 16S rRNA sequence similarity may limit reliable species discrimination.

## Results

### Tick collection, species confirmation, and sequencing overview

**Fig. 1:**
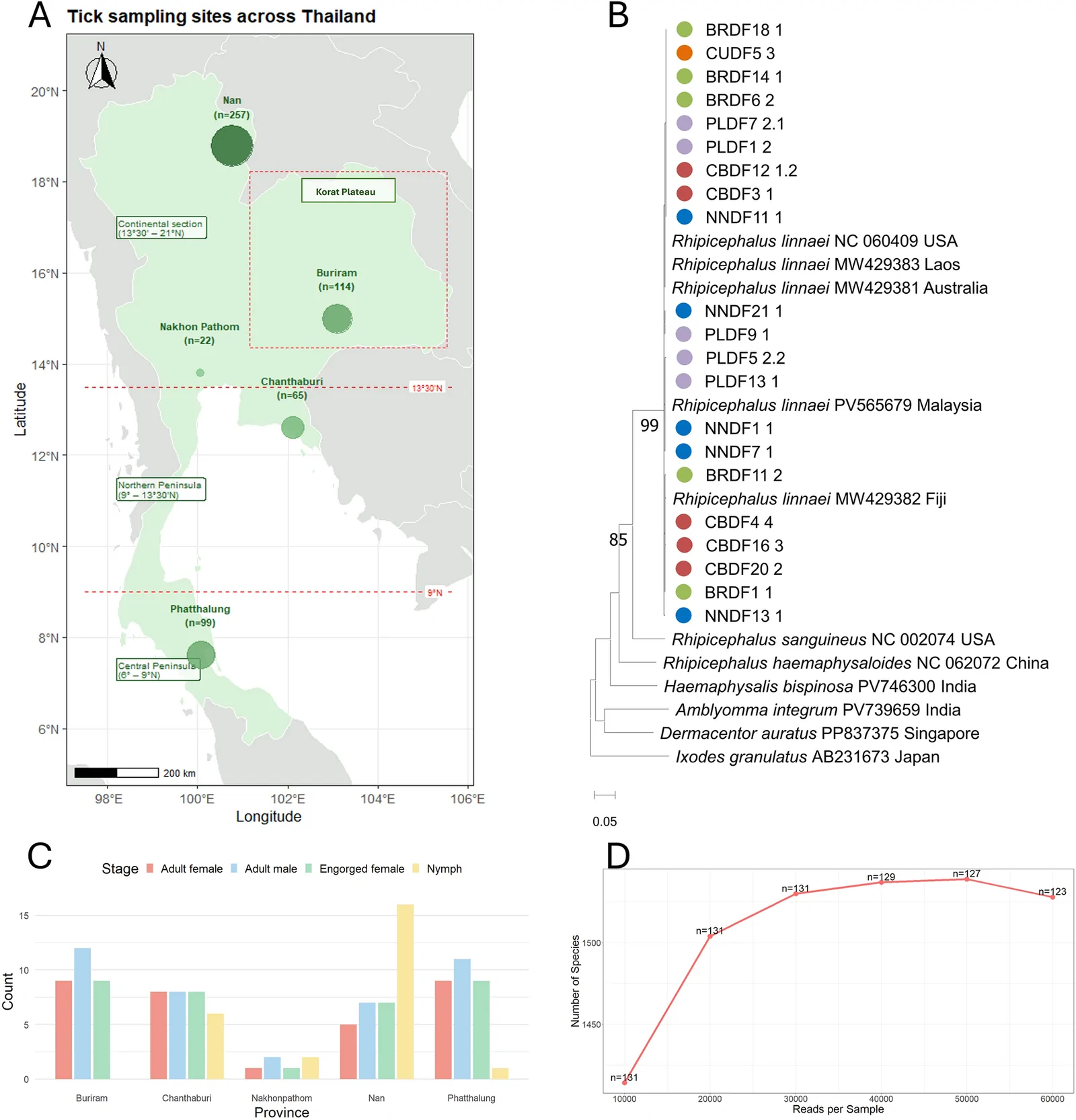
**A** Geographic sampling sites for tick collection. The red dotted lines denote the northern, central, southern, and northeastern regions used for sampling; **B** phylogenetic tree generated from the COX1 sequences from 21 *R. linnaei* from this study and 11reference strains from *Rhipicephalus* and other tick species. The tip label colours indicate that samples were collected from the same location. The scale bar denotes evolutionary distance; **C** number of tick specimens by developmental stage and province subjected to 16S rRNA full-length sequencing; **D** rarefaction curve showing the number of distinct species detected in the dataset as subsampling reads. The numbers denote the number of samples included for each read threshold, which decreases as samples with fewer reads are removed from the analysis

A total of 131 *R. linnaei* ticks were collected from domestic dogs across five provinces in Thailand, including Nan, Chanthaburi, Phatthalung, Buriram, and Nakhonpathom between 2024 and 2025 (Fig. 1A). Tick specimens included both adult and nymphal stages and represented male and female ticks with varying blood-feeding statuses (Fig. 1C). All specimens examined in this study shared identical morphological characteristics and were morphologically assigned to the *R. sanguineus* sensu lato complex. Phylogenetic analysis of 21 systematically selected specimens based on the mtCOI gene assigned all sequenced specimens to *R. linnaei*. Given the concordance between morphological and molecular findings, the tick specimens investigated in this study are referred to as *R. linnaei* throughout the manuscript (Fig. 1B).

Full-length bacterial 16S rRNA sequencing using Oxford Nanopore Technology generated high-quality sequencing data across all samples. Following quality filtering, adapter trimming, and rarefaction at 50,000 reads per sample, 127 samples were retained for downstream microbiome analyses. Rarefaction analysis demonstrated that 50,000 reads were an adequate sequencing depth for characterisation of bacterial community diversity across most samples (Fig. 1D).

### Contamination control and filtering

To minimise the impact of low-abundance sequencing artefacts and potential barcode cross-talk, taxonomic assignments detected at very low relative abundance were interpreted cautiously. A minimum relative abundance threshold of 0.0001 (0.01%), consistent with the default EMU pipeline settings, was applied to all samples. This threshold is relative to sequencing depth; for instance, in a sample with 200,000 reads, taxa represented by fewer than 20 reads fall below the cutoff, whereas in a sample with 1000 reads, taxa with ≥10 reads exceed the threshold and are retained.

Taxa consistently detected at very low abundance across multiple samples were considered likely attributable to background contamination, barcode leakage, or sequencing artefacts. Negative extraction and PCR controls, where available, were assessed during downstream analysis. Putative pathogenic taxa identified solely from low-abundance 16S rRNA gene assignments were not considered confirmatory evidence of infection.

### Overall bacterial community composition

**Fig. 2:**
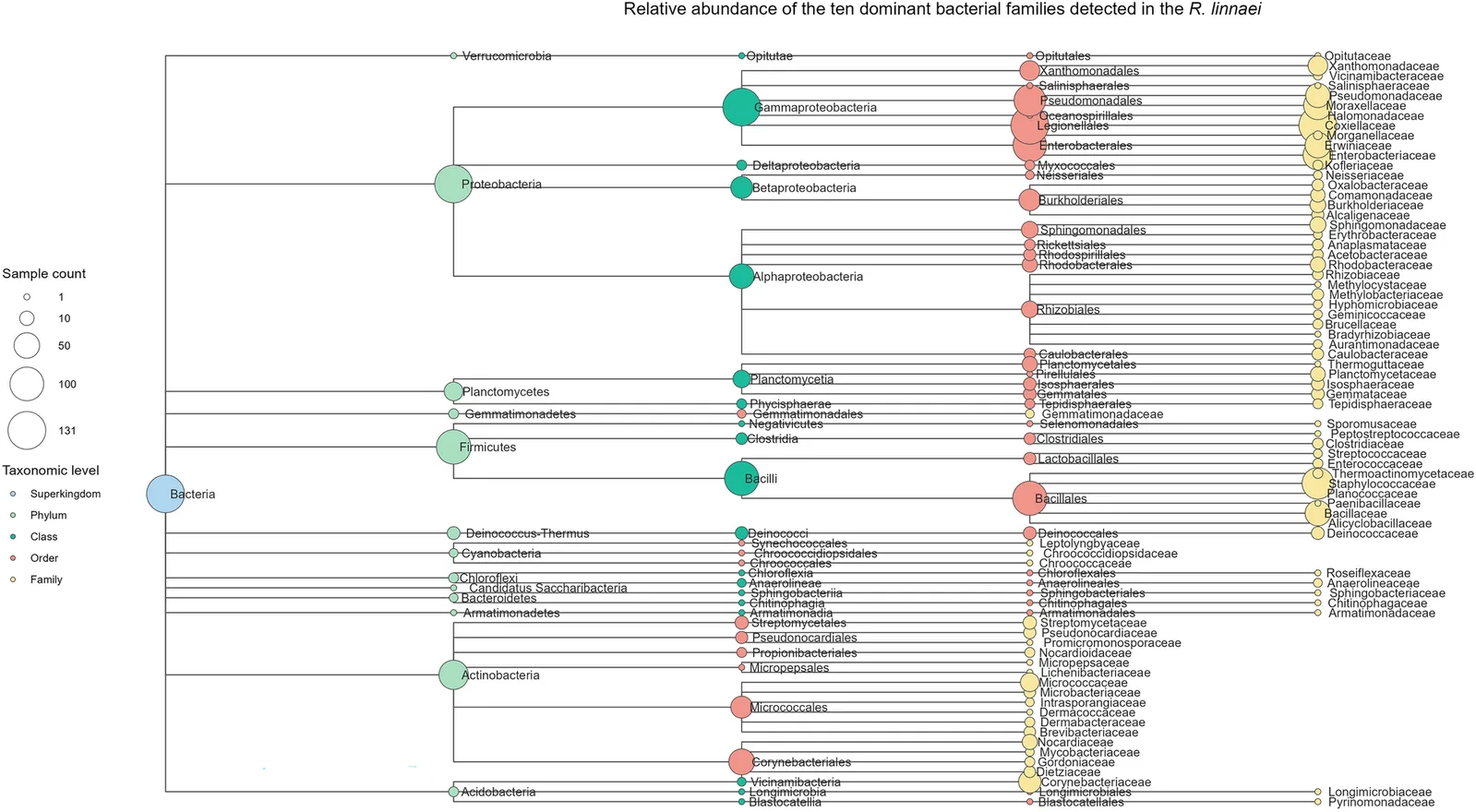
Relative abundance of the ten dominant bacterial families detected in *R. linnaei* samples. Circles represent the number of samples in which each OTU was observed

The microbiome of *R. linnaei* was dominated by *Candidatus* Coxiella mudrowiae, which was detected in all tick samples (100%) (Table 2). Relative abundance analysis demonstrated that bacterial communities were largely composed of members of the families Coxiellaceae, Bacillaceae, Enterobacteriaceae, Pseudomonadaceae, Moraxellaceae, and Staphylococcaceae (Fig. 2). Bacillus species were detected in 72.5% of tick samples, while members of the genera *Enterobacter*, *Acinetobacter*, *Escherichia*, and *Klebsiella* were identified at variable frequencies across sampling regions (Table 2).

**Table 2:** Selected bacterial taxa of potential veterinary or environmental relevance detected in *R. linnaei* across Thailand, based on 16S rRNA full-length sequencing using Oxford Nanopore Technology

Bacterial pathogens	*R. linnaei* in Thai provinces (*n*=131)						
	Nan (*n*=35)	Chanthaburi (*n*=30)	Phatthalung (*n*=30)	Buriram (*n*=30)	Nakhonpathom (*n*=6)	Total	%
Tick-borne disease							
*Anaplasma* spp. (closest match *A. phagocytophilum*)	1	0	1	0	0	2	1.5
*Ehrlichia* spp. (closest match *E. canis*)	0	1	0	1	0	2	1.5
*Candidatus* Coxiella mudrowiae	35	30	30	30	6	131	100
Selected bacterial taxa of potential clinical or environmental relevance [32]							
*Acinetobacter* spp.	34	5	7	6	0	52	39.4
*Citrobacter species*	8	9	1	4	0	22	16.7
*Escherichia/Shigella* spp.	34	21	9	12	4	80	60.6
*Escherichia coli*	8	8	12	14	3	45	34.9
*Klebsiella* spp.	30	4	3	3	0	40	30.3
*Pseudomonas* spp.	8	7	5	7	1	28	22.0
*Staphylococcus* spp.	0	0	1	1	0	2	1.5

The relative abundance of dominant bacterial taxa varied across geographical regions and ecological groupings. Ticks collected from Nan and Chanthaburi demonstrated comparatively greater representation of Enterobacteriaceae-associated taxa, whereas samples from Phatthalung and Buriram demonstrated greater relative abundance of Bacillaceae-associated taxa.

### Alpha diversity analyses

**Fig. 3:**
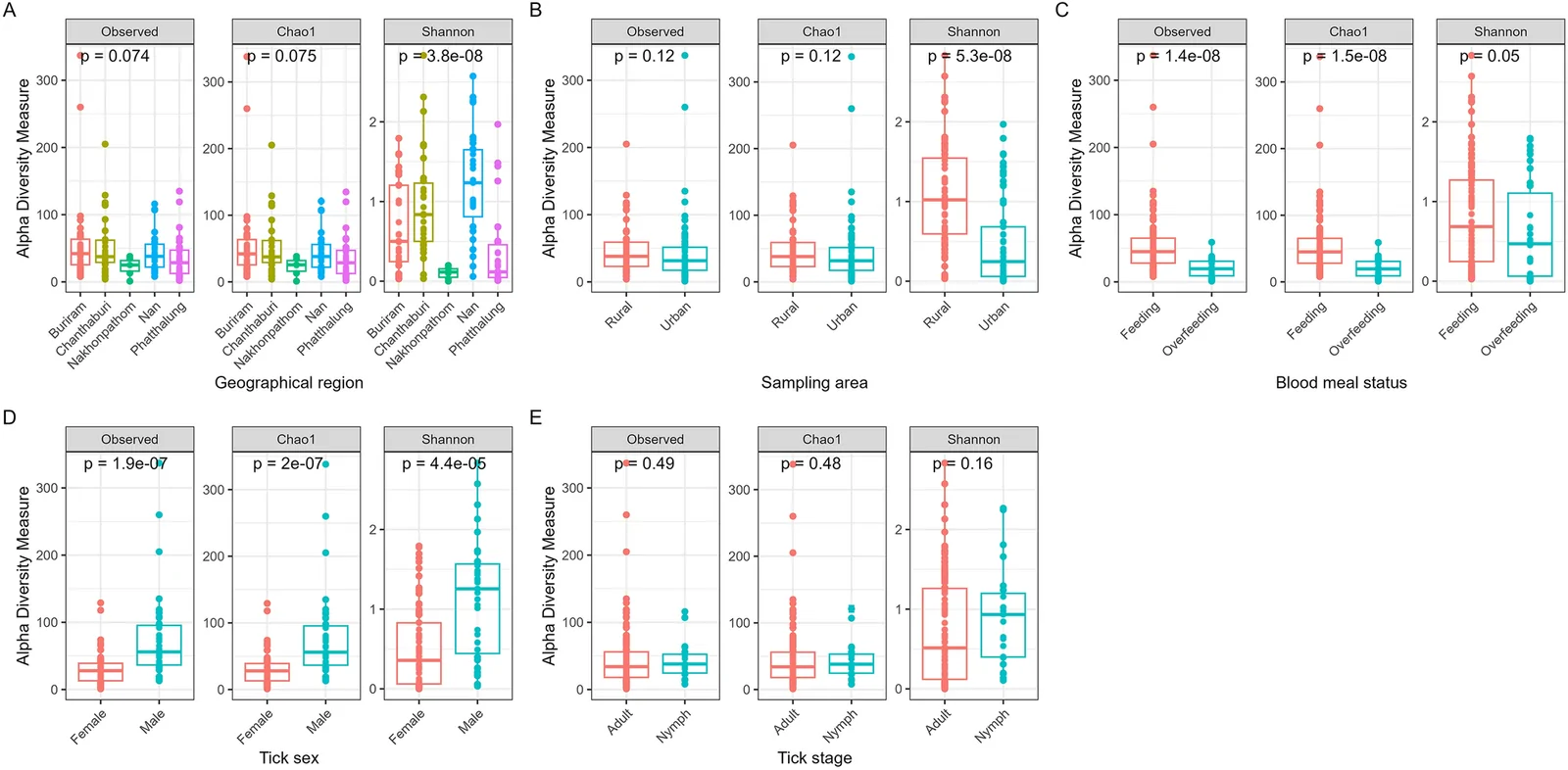
Alpha diversity of bacteria compared across **A** geographical regions; **B** sampling area; **C** blood meal status; **D** tick sex; **E** tick stages. Boxes represent the median and interquartile range

Alpha diversity analyses revealed significant differences in bacterial richness and diversity across geographical regions, sampling areas, and blood-feeding status (Table 1, Fig. 3). Ticks collected from Nan and Chanthaburi showed greater microbial diversity than those collected from Nakhonpathom and Phatthalung. Shannon diversity indices were highest in samples from Nan (median 1.24, Q1–Q3: 0.81–1.65) and lowest in samples from Nakhonpathom (median 0.12, Q1–Q3: 0.05–0.16).

Ticks collected from rural environments demonstrated significantly greater alpha diversity compared with ticks collected from urban settings (*P* ≤ 0.001). Similarly, feeding ticks exhibited greater bacterial richness and diversity than engorged ticks (*P* = 0.05). Male ticks demonstrated greater observed richness and Shannon diversity compared with female ticks, and were statistically significant following multiple comparison correction. No significant differences in alpha diversity were observed between adult and nymphal stages.

### Beta diversity analyses

**Fig. 4:**
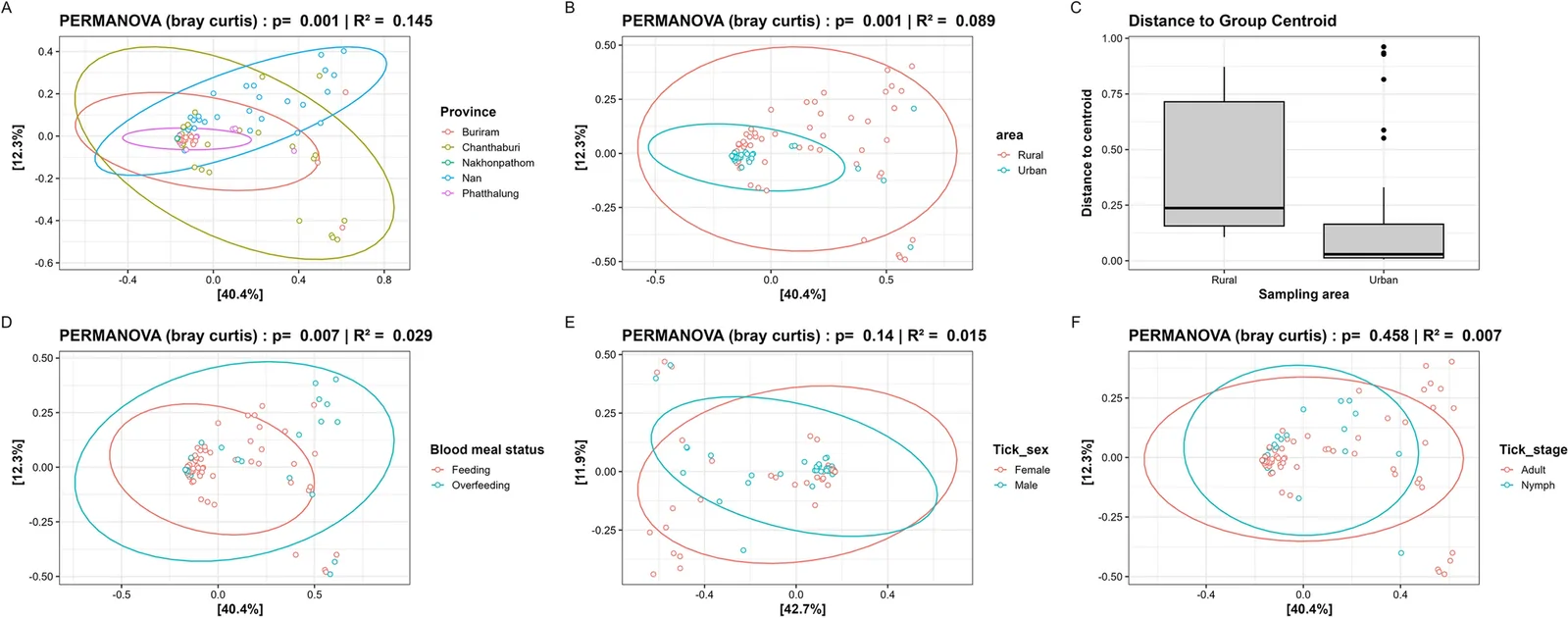
Beta diversity was assessed using principal coordinates analysis (PCoA) based on Bray–Curtis dissimilarity. Ellipses indicate the 95% confidence intervals for each region. Points are coloured by group, and shorter distances between points reflect greater similarity in species composition. **A** Geographical regions; **B** sampling area; **C** beta dispersion of sampling area; **D** blood meal status; **E** tick sex; **F** tick stages

Principal coordinates analysis based on Bray–Curtis dissimilarity revealed significant differences in bacterial community composition across geographical region, sampling area, blood-feeding status, sex, and stage (Fig. 4). Blood meal status was associated with differences in community composition (*P* = 0.007, R^2^ = 0.029, *F* = 3.66), with no significant dispersion effect *P* = 0.055). Similarly, sex had a significant effect on community composition (*P* = 0.009, R^2^ = 0.017, *F* = 3.48), with no evidence of dispersion heterogeneity (*P* = 0.258). Blood meal was associated with directional differences in the microbial community composition, with overfeeding status showing greater microbiome variability.

Significant differences in bacterial community composition were observed across both geographical regions and sampling areas (*P* = 0.001, Table 1). Geographical region explained 14.5% of the total variation (PERMANOVA: *F* = 5.16, *P* = 0.001), while sampling area accounted for 8.9% of the variation (*F* = 12.23, *P* = 0.001). Notably, significant differences in multivariate dispersion were observed between the two groupings (*P* = 0.001; Table 1). For the sampling area, a pronounced variance effect was detected (*F* = 34.02), indicating distinct within-group heterogeneity between rural and urban. In contrast, life stage did not significantly influence community composition (PERMANOVA, *p* = 0.458, R^2^ = 0.007, *F* = 1.48), and dispersion did not differ significantly (*P* = 0.358). Similarly, sex was not significant effect on community composition (*P* = 0.14, R² = 0.015, F = 3.48), with no evidence of dispersion heterogeneity (*P* = 0.258).The tick sex and developmental stage were not significantly associated with beta diversity in this dataset.

### Differential abundance analysis

**Fig. 5:**
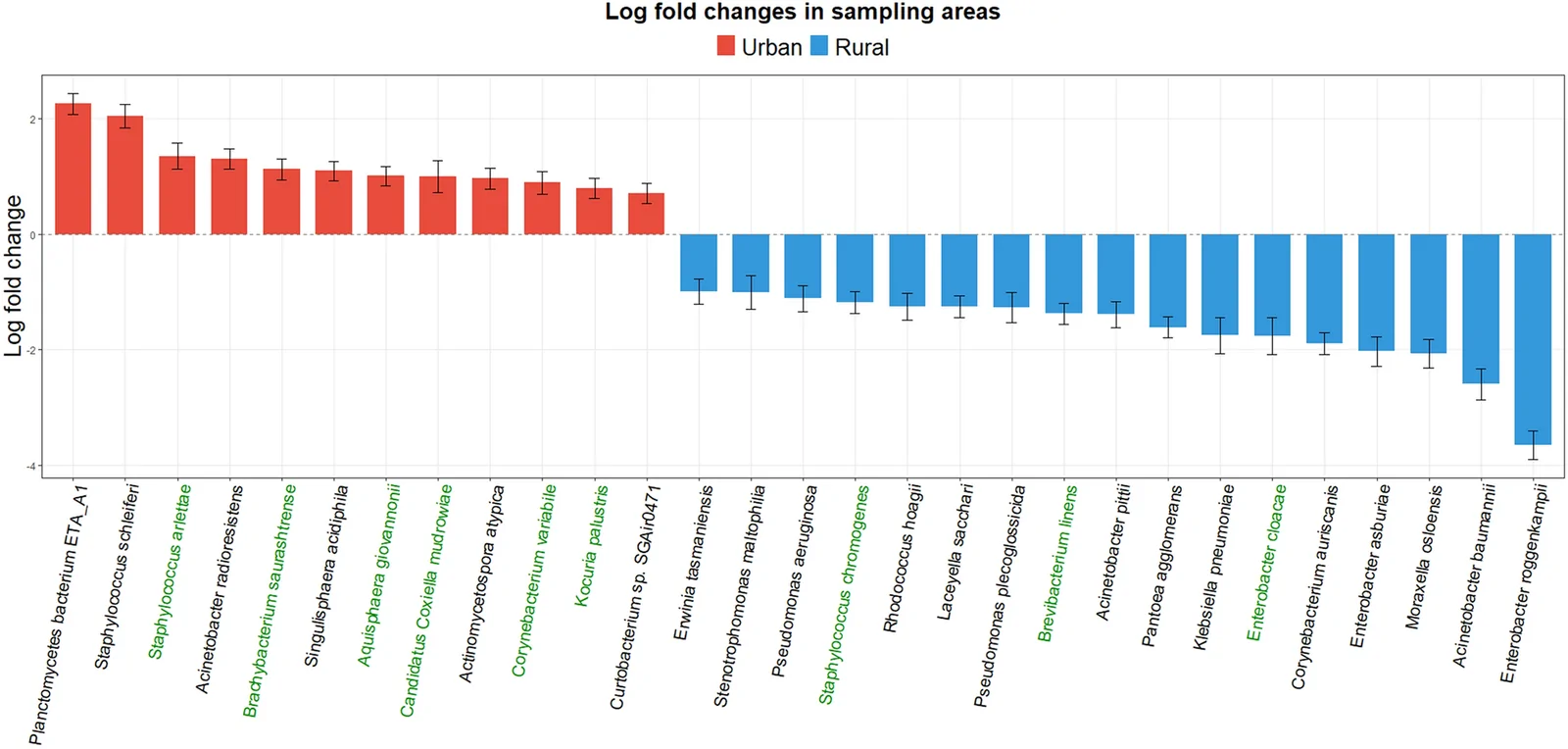
Differential abundance analysis of bacterial taxa in *R. linnei* using ANCOM-BC2. Red bars indicate taxa with higher relative abundance in urban ticks, while blue bars indicate taxa enriched in rural ticks

Differential abundance analysis using ANCOM-BC2 identified several bacterial taxa significantly associated with ecological and host-related variables (Fig. 5). Urban-associated ticks demonstrated relative enrichment of selected Enterobacteriaceae-associated taxa, while rural-associated ticks showed comparatively greater abundance of environmental and soil-associated bacterial groups. Feeding ticks demonstrated enrichment of several bacterial taxa not commonly observed in overfed or engorged ticks, suggesting that blood-feeding status may substantially influence microbial community composition. Geographical variation in bacterial abundance patterns was also observed, with specific taxa demonstrating regional enrichment across the sampled provinces. However, the magnitude of these differences varied between taxa and ecological groupings.

### Detection of bacterial taxa of potential veterinary relevance

Several bacterial taxa of potential veterinary and medical relevance were detected at low frequency within tick samples (Table 2). Sequence assignments corresponding to *Anaplasma* spp., with the closest match to *A. phagocytophilum*, were identified in two tick samples (1.5%). Sequence assignments corresponding to *Ehrlichia* spp., with the closest match to *E. canis*, were similarly identified (1.5%). Additional bacterial genera, including *Acinetobacter*, *Klebsiella*, *Pseudomonas*, *Escherichia/Shigella*, and *Staphylococcus,* were identified at variable abundance across samples.

The distribution of these taxa varied geographically, with *Acinetobacter baumannii* most frequently detected in samples from Nan and *Escherichia coli* more commonly identified in ticks collected from Phatthalung and Buriram. However, these findings should be interpreted with caution, as taxonomic assignment was based solely on 16S rRNA sequencing, and no confirmatory molecular testing was performed.

## Discussion

This study characterised the bacterial microbiome of *Rhipicephalus linnaei* collected from dogs across multiple geographical regions of Thailand using full-length 16S rRNA sequencing on an ONT platform. The findings indicate that geographical location, sampling environment, and blood-feeding status were associated with significant differences in bacterial community diversity and composition. The tick sex and developmental stage had comparatively weaker effects. Overall, the results support the concept that both environmental and host-associated factors contribute to shaping the microbiome of *R. linnaei* in tropical settings.

The microbiome of *R. linnaei* in this study was dominated by *Candidatus* Coxiella mudrowiae, which was identified in all tick specimens. Coxiella-like endosymbionts are widely recognised as common and often dominant members of the microbiome of *Rhipicephalus* ticks. They are thought to play important roles in tick physiology and nutritional supplementation, particularly through the synthesis of B vitamins and other essential metabolites [11, 12]. The widespread detection of *Candidatus* Coxiella in the present study is therefore consistent with observations from previous investigations of ixodid tick microbiomes in both tropical and temperate regions [9, 11]. The apparent ubiquity of this endosymbiont further supports the hypothesis that symbiotic bacterial relationships are central to the biology and survival of *Rhipicephalus* ticks.

Geographical region was associated with significant differences in bacterial alpha and beta diversity, although the PERMANOVA effect size was modest. These findings suggest that geographical and ecological factors contribute to variation in microbial community structure but likely account for only a portion of overall microbiome variability. Differences in environmental microbial exposure, climatic conditions, host-associated bacterial communities, local habitat characteristics, and anthropogenic influences may all contribute to the observed regional variation [14, 16]. Similar geographical structuring of tick-associated microbiomes has been reported in studies of *Ixodes* and *Rhipicephalus* species from other regions [16]. The relatively low R^2^ values observed in the present study indicate that additional unmeasured variables, including host-level factors and microenvironmental conditions, are also likely to influence microbiome composition.

Differences in bacterial diversity were also observed between ticks collected from urban and rural environments. Urban-associated ticks showed lower bacterial diversity than rural ticks, potentially reflecting differences in environmental exposure, host movement patterns, habitat diversity, and antimicrobial exposure associated with urban ecosystems. Urbanisation has been increasingly associated with altered microbial ecology across a range of arthropod vectors and animal hosts [30]. Reduced microbial diversity in urban environments may reflect ecological homogenisation and altered environmental microbial reservoirs. However, the mechanisms underlying these observations remain incompletely understood and warrant further investigation.

Blood-feeding status was another important factor associated with variation in the microbial community. Feeding ticks demonstrated significantly greater bacterial richness and diversity compared with overfed or engorged ticks. Blood meals may alter tick-associated microbial communities through several mechanisms, including the introduction of host-derived microorganisms, physiological changes within the tick gut, altered nutrient availability, and selective microbial proliferation during digestion [18]. Previous studies have similarly demonstrated that blood feeding can substantially influence microbiome composition in hematophagous arthropods, including ticks and mosquitoes [14, 17]. The present findings therefore support the concept that host blood meals represent an important ecological driver of microbial dynamics within *R. linnaei*.

In contrast, tick sex and developmental stage had comparatively limited effects on microbial diversity and community structure. Although male ticks demonstrated higher alpha diversity when compared with female ticks, the difference between bacterial community diversity was not statistically significant after correction for multiple comparisons. Similarly, nymphs and adults showed broadly similar microbial community profiles. Previous studies investigating sex- and stage-associated microbiome differences in ticks have reported variable findings, suggesting that these factors may exert species-specific or environmentally dependent effects [15, 16]. The relatively weak associations observed in this study may indicate that environmental exposure and feeding status have a greater influence on the microbiome of *R. linnaei* than intrinsic biological characteristics.

Several bacterial taxa of potential veterinary and medical relevance were identified at low frequency, including *Anaplasma phagocytophilum* and *Ehrlichia canis*. However, these findings should be interpreted cautiously. Detection was based solely on 16S rRNA sequencing, which does not confirm bacterial viability, pathogenicity, or active infection within the tick. Furthermore, some bacterial taxa identified at low abundance may represent environmental contamination, transient surface-associated organisms, reagent contamination or taxonomic misclassification. Additional confirmatory approaches, including targeted PCR assays, quantitative molecular methods, and metagenomic sequencing beyond the 16S gene, would be required to validate these observations.

The use of ONT full-length 16S rRNA sequencing was a strength of this study, enabling improved taxonomic resolution compared with short-read approaches targeting partial 16S regions. Full-length sequencing may improve species-level assignment, facilitate more comprehensive characterisation of arthropod-associated microbial communities [19, 20] and reduce bias associated with specific 16S primer sets [31]. 16S rRNA sequencing is also cost-effective and enables the study of larger sample sizes than shotgun metagenomic approaches. Nevertheless, several limitations should be acknowledged. First, 16S rRNA sequencing cannot distinguish viable from non-viable bacteria and has limited ability to discriminate among closely related taxa within certain bacterial groups. Second, microbiome studies involving low-biomass samples such as ticks are particularly vulnerable to contamination introduced during DNA extraction, PCR amplification, and sequencing workflows. Finally, the cross-sectional nature of the study limits interpretation regarding temporal microbiome dynamics and causal ecological relationships.

This study has several limitations. Interpretation of low-abundance taxa should be approached with caution, particularly in low-biomass microbiome studies, where reagent contamination, barcode cross-talk, and sequencing artefacts may lead to spurious taxonomic assignments. This is especially relevant for taxa detected in only a small number of samples and at low relative abundance.

## Conclusions

In conclusion, this study demonstrated that geography, habitat type, and blood-feeding status are associated with variation in the microbiome of *Rhipicephalus linnaei* in Thailand. The dominance of *Candidatus* Coxiella and the modest but significant ecological structuring of bacterial communities highlight the importance of both environmental and host-associated factors in shaping tick-associated microbiomes. These findings contribute to the limited body of data describing tick microbiomes in Southeast Asia and provide a foundation for future investigations examining the functional significance of microbial communities in tick biology and vector competence.

## Data Availability

The COXI gene sequences from ticks have been deposited in the NCBI database under accession numbers PZ440876–PZ440896. Raw 16S rRNA sequencing reads generated using the GridION platform are available in the NCBI Sequence Read Archive (SRA) under BioProject PRJNA1469838.
